# Preserving blood-retinal barrier integrity: a path to retinal ganglion cell protection in glaucoma and traumatic optic neuropathy

**DOI:** 10.1186/s13619-025-00228-y

**Published:** 2025-04-02

**Authors:** Lai-Yang Zhou, Zhen-Gang Liu, Yong-Quan Sun, Yan-Zhong Li, Zhao-Qian Teng, Chang-Mei Liu

**Affiliations:** 1 Key Laboratory of Organ Regeneration and Reconstruction, Institute of Zoology, Chinese Academy of Sciences, Beijing, 100101 China; 2 Savaid Medical School, University of Chinese Academy of Sciences, Beijing, 100049 China; 3 Institute for Stem Cell and Regeneration, Chinese Academy of Sciences, Beijing, 100101 China; 4 Beijing Institute for Stem Cell and Regenerative Medicine, Beijing, 100101 China; 5 Department of Orthopaedics, China-Japan Union Hospital of Jilin University, Changchun, 130033 China

**Keywords:** Retinal ganglion cell, Blood-Retinal Barrier, Glaucoma, Traumatic Optic Neuropathy, Microenvironment, Neurovascular Unit, Neuroprotection

## Abstract

Retinal ganglion cells (RGCs) are the visual gateway of the brain, with their axons converging to form the optic nerve, making them the most vulnerable target in diseases such as glaucoma and traumatic optic neuropathy (TON). In both diseases, the disruption of the blood-retinal barrier(BRB) is considered an important mechanism that accelerates RGC degeneration and hinders axon regeneration. The BRB consists of the inner blood-retinal barrier (iBRB) and the outer blood-retinal barrier (oBRB), which are maintained by endothelial cells(ECs), pericytes(PCs), and retinal pigment epithelial (RPE), respectively. Their functions include regulating nutrient exchange, oxidative stress, and the immune microenvironment. However, in glaucoma and TON, the structural and functional integrity of the BRB is severely damaged due to mechanical stress, inflammatory reactions, and metabolic disorders. Emerging evidence highlights that BRB disruption leads to heightened vascular permeability, immune cell infiltration, and sustained chronic inflammation, creating a hostile microenvironment for RGC survival. Furthermore, the dynamic interplay and imbalance among ECs, PCs, and glial cells within the neurovascular unit (NVU) are pivotal drivers of BRB destruction, exacerbating RGC apoptosis and limiting optic nerve regeneration. The intricate molecular and cellular mechanisms underlying these processes underscore the BRB's critical role in glaucoma and TON pathophysiology while offering a compelling foundation for therapeutic strategies targeting BRB repair and stabilization. This review provides crucial insights and lays a robust groundwork for advancing research on neural regeneration and innovative optic nerve protective strategies.

**Graphical Abstract:**

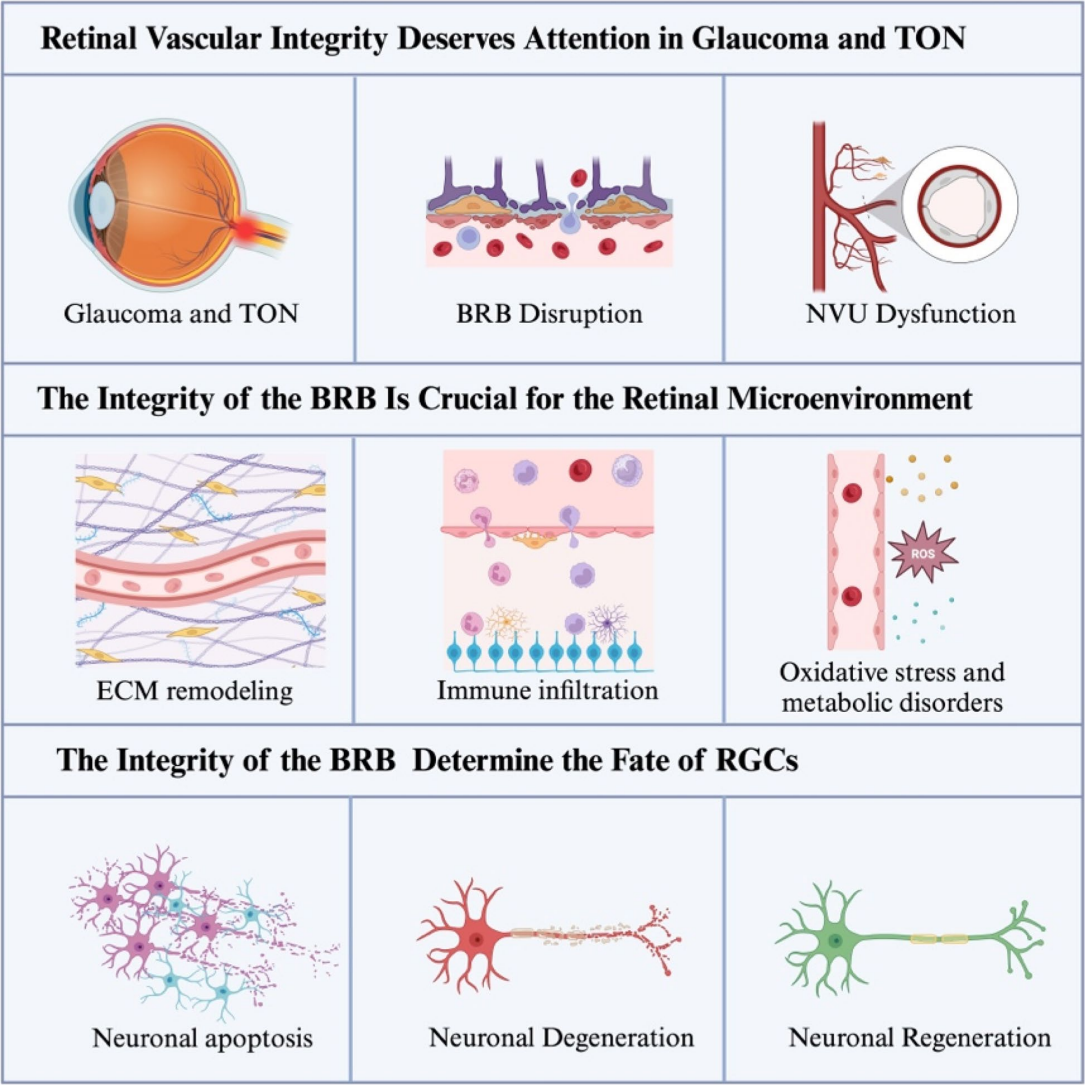

## Background

RGCs are the only neurons in the retina that transmit visual information to the brain through their long axons, which converge to form the optic nerve (Laha et al. 2017; Tian et al. 2022). This unique connectivity makes the optic nerve highly susceptible to external damage, as seen in diseases such as glaucoma and TON (Li et al. 2022; Wang et al. 2023a, b). RGCs are particularly vulnerable to mechanical and ischemic insults, and upon exposure to such stressors, a cascade of pathological events is triggered, including axonal injury, RGC degeneration, metabolic dysregulation, and retinal inflammation (Varadarajan et al. 2022; Jacobi et al. 2022). Glaucoma represents a chronic optic nerve injury, while TON involves acute optic nerve damage (Williams et al. 2017; Zhou et al. 2023). Researchers often employ the optic nerve crush (ONC) model or magnetic bead injection model in mice to explore the molecular mechanisms of these injuries (Wang et al. 2023a, b; Li et al. 2024a, b). Ultimately, the death of RGCs leads to irreversible vision loss, highlighting the urgent need to investigate the mechanisms underlying RGC degeneration and to develop effective therapeutic strategies (Serger et al. 2022; Abbas et al. 2022).

While the primary focus of research has often been on the intrinsic vulnerability of RGCs, it is becoming increasingly evident that their survival and function are intricately linked to the retinal microenvironment (He and Jin 2016; Au and Ma 2022; Benhar et al. 2023). The retina relies on a dual blood supply system—the central retinal artery and the choroidal capillary plexus—which ensures the high metabolic demand of the tissue is met (Toma et al. 2024). These vascular networks not only provide oxygen and nutrients but also form the anatomical foundation of BRB, a crucial structure for maintaining the homeostasis of the retinal microenvironment (Mills et al. 2021). The BRB is subdivided into the inner BRB (iBRB) and outer BRB (oBRB), both of which have unique physiological roles (Langen et al. 2019). Recent studies emphasize the BRB's critical function in regulating nutrient exchange, immune modulation, and oxidative stress, which collectively safeguard RGC survival and function (Toma et al. 2024; Ren et al. 2024; Peng et al. 2024).

Although the role of the BRB has been extensively studied in systemic vascular diseases, such as diabetic retinopathy, its involvement in diseases like glaucoma and optic nerve injury has received comparatively less attention (Jo et al. 2019; Chen et al. 2019). Emerging evidence suggests that BRB dysfunction in these conditions leads to increased vascular permeability, immune cell infiltration, and inflammatory responses, all of which exacerbate RGC degeneration and hinder optic nerve regeneration (McMenamin et al. 2019; Tomkins-Netzer et al. 2024; Passino et al. 2024). The breakdown of the BRB not only disrupts retinal homeostasis but also creates a hostile immune microenvironment for RGCs (Xie et al. 2021; Habibi-Kavashkohie et al. 2023). These findings underscore the need for further research into the mechanisms of BRB disruption and its therapeutic potential in mitigating RGC loss in glaucoma and TON (Song et al. 2016; Li et al. 2022).

This review aims to summarize the structural and functional characteristics of the BRB, explore its mechanisms of disruption in glaucoma and TON, and evaluate its impact on RGC fate. Furthermore, it integrates the latest foundational and preclinical research to discuss BRB-targeted therapeutic strategies, offering a theoretical basis for improving treatments for glaucoma and TON.

## Function and structure of the BRB

**Fig. 1:**
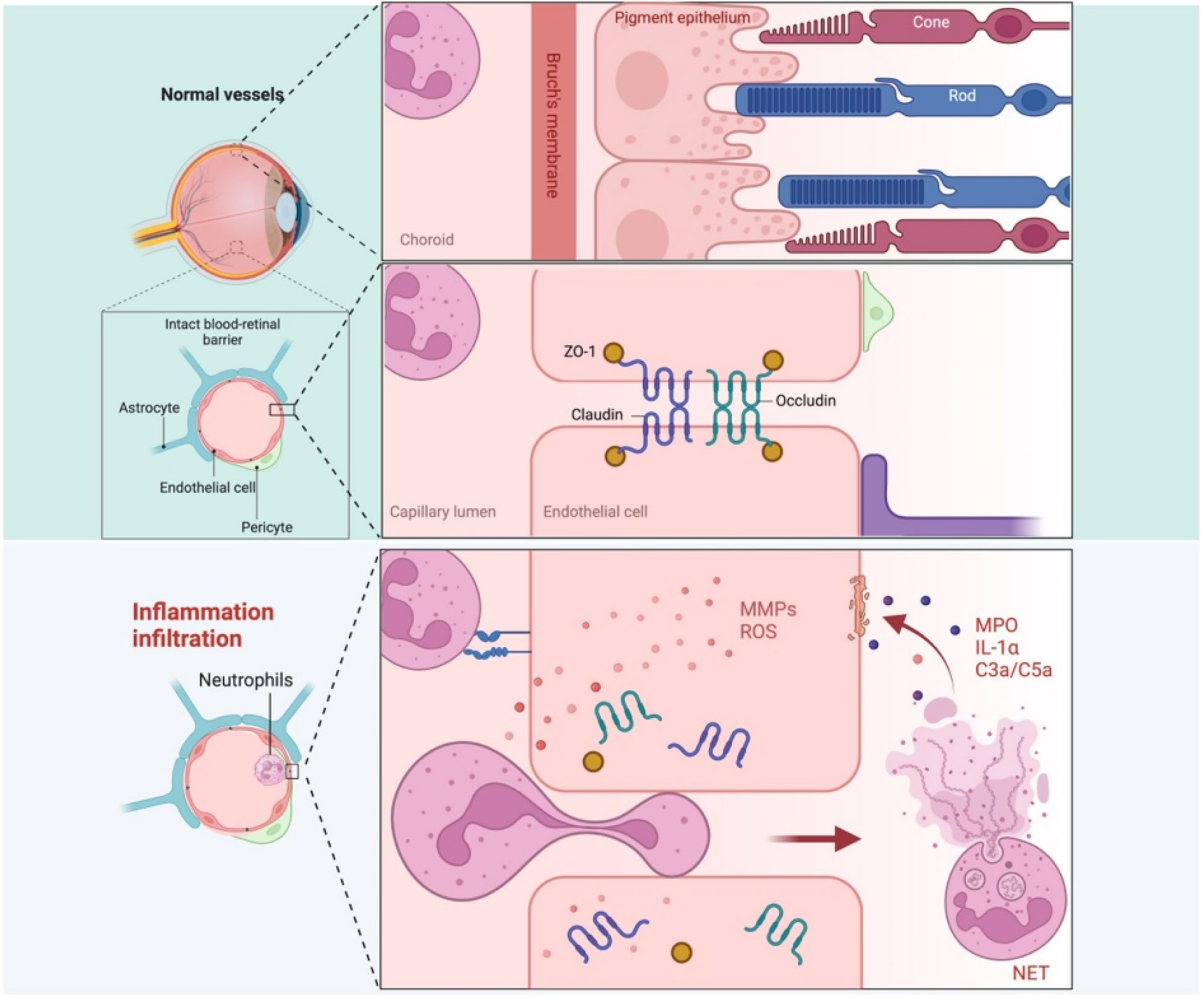
Blood-retinal barrier (BRB) in health and disease. Healthy state (top panel): The intact BRB consists of the iBRB, formed by endothelial cells with tight junctions (ZO-1, claudin, occludin), supported by pericytes and astrocytes, and oBRB, maintained by RPEs. Together, these structures regulate nutrient transport, immune protection, and oxidative stress, ensuring retinal homeostasis. Pathological state (bottom panel): BRB disruption in disease conditions, such as glaucoma or optic nerve injury, is characterized by neutrophil infiltration and the release of inflammatory mediators (e.g., ROS, MMPs, MPO, IL-1α, C3a/C5a). These factors degrade tight junction proteins, increase vascular permeability, and destabilize the retinal microenvironment, leading to neuronal degeneration and impaired retinal function

The BRB is a highly specialized vascular structure essential for maintaining retinal homeostasis and protecting RGCs from pathological stress (O’Leary and Campbell 2023). It is composed of two distinct yet interconnected components: iBRB and oBRB, which work in concert to preserve the retina's metabolic and immune stability (Fig. 1) (Díaz-Coránguez et al. 2017). These barriers regulate the selective exchange of nutrients, oxygen, and metabolic byproducts while preventing the infiltration of harmful substances, including immune cells and pro-inflammatory mediators, into the retinal microenvironment (Fields et al. 2020; Xia et al. 2021).

The inner BRB is formed by retinal ECs, which are tightly interconnected by specialized junctional complexes, including tight junction proteins such as occludins, claudins, and zonula occludens-1 (ZO-1)(Antonetti et al. 2021). These structures restrict paracellular permeability and play a key role in maintaining the immune-privileged status of the retina. ECs are supported by PCs, which are closely associated with the endothelial basement membrane and provide structural and functional stability to the retinal vasculature (Díaz-Coránguez et al. 2017; Gurler et al. 2023). PCs regulate endothelial behavior through paracrine signaling and contribute to the integrity of the iBRB by secreting growth factors such as platelet-derived growth factor- beta (PDGF-β) and transforming growth factor-beta (TGF-β)(Rudraraju, Narayanan, and Somanath 2020; Huang 2020). Together, ECs and PCs form the NVU, which dynamically coordinates vascular stability with neuronal and glial activity, ensuring the high metabolic demands of RGCs are met (Wareham and Calkins 2020).

The outer BRB is maintained by RPE, which form a selective barrier between the neural retina and the choroidal vasculature (Kaufmann and Han 2024). RPE perform critical functions, including the transport of nutrients such as glucose and fatty acids to the photoreceptors, the removal of metabolic waste, and the recycling of visual cycle components (Sharma et al. 2020). Their intercellular tight junctions, similar to those in the iBRB, ensure selective permeability while restricting the entry of inflammatory cells and large macromolecules from the systemic circulation (Lakkaraju et al. 2020). The RPE also plays a pivotal role in modulating oxidative stress and regulating lipid metabolism, which are essential for maintaining the stability of the outer retinal environment (Gabrielle 2022).

These dual barriers—the iBRB and oBRB—operate synergistically to regulate retinal homeostasis (Fig. 1) (Zhou et al. 2024a). The iBRB interacts closely with the NVU, which includes glial cells such as astrocytes and Müller cells (Abcouwer et al. 2021). Astrocytic endfeet envelop the retinal capillaries, reinforcing tight junction integrity and contributing to ionic and water homeostasis through channels such as aquaporin-4 (AQP4) (Wang et al. 2020; Shinozaki et al. 2023). Similarly, Müller cells, which span the entire thickness of the retina, provide metabolic support to neurons and regulate extracellular glutamate levels, ensuring neuronal survival and function (Palazzo et al. 2022). The oBRB, on the other hand, maintains a stable microenvironment for photoreceptors and indirectly supports RGC health by preserving overall retinal homeostasis (Gabrielle 2022).

Under physiological conditions, the BRB is remarkably effective at shielding the retina from systemic fluctuations and pathological stressors (Gonçalves et al. 2016; Hynynen 2024). However, its disruption is a hallmark of many retinal diseases, including glaucoma and TON (Alarcon-Martinez et al. 2023). In these conditions, BRB dysfunction is associated with increased vascular permeability, immune cell infiltration, and chronic inflammation, all of which contribute to the destabilization of the retinal microenvironment and the progressive degeneration of RGCs (Qijun et al. 2019; Antonetti et al. 2021). Understanding the structural and functional roles of the BRB in health and disease is essential for identifying therapeutic targets aimed at preserving its integrity and mitigating retinal neurodegeneration.

## The role of the NVU in supporting iBRB integrity

The NVU, composed of ECs, PCs, astrocytes, Müller cells, and microglia, is essential for maintaining iBRB integrity under physiological conditions (Chow et al. 2020). This tightly coordinated system ensures the metabolic, structural, and immune stability required for normal retinal function, thereby safeguarding RGCs and supporting their high energy demands (Liang et al. 2023).

ECs, as the central structural component of the iBRB, form a highly selective barrier through tight junction proteins such as occludins, claudins, and ZO-1 (Haas et al. 2022). These tight junctions strictly regulate the exchange of nutrients, oxygen, and metabolic byproducts, while preventing the entry of harmful substances like inflammatory mediators (Varadarajanet al. 2019). ECs also play a pivotal role in preserving immune privilege by suppressing the expression of adhesion molecules, thereby minimizing unnecessary immune cell infiltration into the retinal tissue (Pérez-Gutiérrez and Ferrara 2023).

PCs, closely associated with ECs, are critical for iBRB stability (Alarcon-Martinez et al. 2020). Through PDGF-B/PDGFRβ signaling, PCs support EC behavior, enhance tight junction integrity, and stabilize the retinal vasculature (Zhu 2024). PCs also regulate capillary blood flow, ensuring precise oxygen and nutrient delivery in response to neuronal activity, which is vital for RGC survival and synaptic function (Alarcon-Martinez et al. 2022).

Astrocytes, concentrated around retinal capillaries and the optic nerve head, reinforce EC tight junctions through their endfeet, contributing to the structural stability of the NVU (Guttenplan et al. 2020). Additionally, astrocytes maintain ionic and water homeostasis via AQP4 channels and secrete neurotrophic factors that support neuronal health, further ensuring a stable retinal microenvironment (Wang et al. 2020).

Müller cells, spanning the entire thickness of the retina, are essential for metabolic and structural support (Cheng et al. 2023). By removing excess glutamate, regulating ionic balance, and providing metabolic substrates, Müller cells actively stabilize the retinal environment and indirectly protect RGCs (Salkar et al. 2024). Their supportive role in connecting vascular and neuronal components is critical for the seamless integration of NVU functions (Soucy et al. 2023).

Microglia, as resident immune cells, play a subtle yet important role in NVU homeostasis under normal conditions (Vecino et al. 2016). By releasing anti-inflammatory cytokines such as IL-10 and TGF-β, microglia help maintain a quiescent immune state and protect iBRB integrity (Phipps et al. 2019; Yun-Jia et al. 2021). They also perform surveillance functions, clearing debris and ensuring an immune-stable microenvironment conducive to retinal health (Borst et al. 2021).

Together, these NVU components form a dynamic, interdependent system that preserves iBRB integrity, providing the retina with the stability necessary for RGC function and overall visual health. By maintaining the delicate balance of metabolic, structural, and immune factors, the NVU plays an indispensable role in retinal physiology.

## Mechanisms of BRB disruption in glaucoma and TON

The BRB is an essential structure for maintaining retinal homeostasis and protecting RGCs from pathological stressors (Monickaraj et al. 2023). Composed of the iBRB, formed by EC and PC, and the oBRB, maintained by RPE, it tightly regulates vascular permeability and immune cell infiltration (Salkar et al. 2024). However, in glaucoma and TON, BRB disruption initiates a cascade of pathological changes—including inflammation, oxidative stress, and vascular instability—that compromise RGC survival, impair axonal regeneration, and exacerbate neurodegeneration (Zhang et al. 2024b). Understanding the molecular mechanisms driving BRB breakdown is critical for identifying therapeutic targets to mitigate these processes.

In glaucoma, sustained intraocular pressure (IOP) elevation imposes mechanical stress on ECs and PCs, resulting in dysregulation of genes associated with extracellular matrix (ECM) remodeling and vascular stability (Reinhard et al. 2021). Specifically, ECs exhibit upregulation of ECM-related genes such as *Col1a1* and *Acta2*, which contribute to ECM remodeling and vascular destabilization, alongside downregulation of nutrient transport-related genes like *Apoe*, further impairing metabolic support to RGCs (Wang et al. 2024). PCs, integral to vascular stability, also undergo pathological changes, including upregulation of inflammatory markers such as *Aqp1* and *Igfbp3* and a reduction in adhesion-related genes like *Tagln* (Yao et al. 2024). These molecular alterations weaken EC-PC interactions, heighten vascular permeability, and increase the retina's susceptibility to inflammatory damage (Yao et al. 2024). Together, these processes drive microvascular instability and accelerate RGC degeneration, a hallmark of glaucoma pathology.

In the ONC model, retinal inflammation following injury triggers robust neutrophil recruitment, exacerbating BRB disruption through the release of reactive oxygen species (ROS), proteases, and neutrophil extracellular traps (NETs)(Jo et al. 2019; McMenamin et al. 2019; Passino et al. 2024). These factors degrade tight junction proteins and ECM components, such as laminin and collagen, significantly increasing vascular permeability and destabilizing the BRB (Ivanova et al. 2019). This disruption intensifies retinal inflammation and compromises RGC survival. Additionally, ONC-induced BRB breakdown is accompanied by significant molecular and cellular changes within the NVU (Syc-Mazurek and Libby 2019). Recent studies have identified astrocyte-mediated glia limitans tightening as a pivotal neuroprotective mechanism in mitigating BRB breakdown (Lefevere et al. 2020). Matrix metalloproteinase-3 (MMP-3) plays a central role in this process by promoting the upregulation of astrocyte-specific tight junction proteins and limiting immune cell infiltration into the retina. In the absence of MMP-3, the glia limitans weakens, resulting in increased leukocyte infiltration, elevated levels of pro-inflammatory cytokines such as IL-1β, and accelerated RGC death. These findings underscore the dual roles of astrocyte-driven protective mechanisms and neutrophil-mediated damage in BRB disruption, emphasizing the importance of preserving NVU integrity to mitigate inflammation-induced retinal degeneration.

Similarly, ischemia–reperfusion (I/R) injury, a hallmark of TON, exacerbates BRB breakdown through oxidative and inflammatory pathways (Heng et al. 2019; Yun-Jia et al. 2021; Kim et al. 2024). Ischemic episodes deprive retinal tissues of oxygen and nutrients, while reperfusion induces a surge of ROS and pro-inflammatory cytokines (Jiang et al. 2020). These ROS directly damage ECs, activate matrix metalloproteinases (MMPs), and degrade ECM components such as laminin and collagen, further destabilizing vascular integrity (Abcouwer et al. 2021). This cascade amplifies immune cell infiltration, perpetuates inflammation, and accelerates RGC degeneration.

Key molecular pathways implicated in BRB dysfunction, including the stromal cell-derived factor-1α (SDF-1α)/C-X-C chemokine receptor type 4 (CXCR4) axis and the PDGF-β/ PDGFRβ signaling pathway, further contribute to vascular instability (Park et al. 2017; Omori et al. 2018). Dysregulation of these pathways disrupts NVU homeostasis, accelerates neuroinflammation, and exacerbates tissue damage, making them attractive targets for therapeutic intervention (Iadecola 2017). Exploring these mechanisms offers valuable insights into the molecular drivers of BRB breakdown and its role in the progression of retinal neurodegenerative diseases.

Understanding the distinct yet overlapping mechanisms contributing to BRB disruption in glaucoma and TON provides a robust framework for developing targeted therapeutic strategies. In the next section, we will delve deeper into how inflammation, oxidative stress, and metabolic dysregulation interact to shape the pathological retinal landscape, further complicating the preservation of RGC integrity and the promotion of neural repair.

## NVU dysfunction in glaucoma and TON

In glaucoma and TON, the breakdown of BRB plays a pivotal role in driving retinal neurodegeneration and RGC death (Wareham and Calkins 2020). The dysfunction of the NVU exacerbates this pathological process by disrupting the coordinated interactions between its key components—ECs, PCs, astrocytes, Müller cells, and microglia—creating a vicious cycle of damage and inflammation (Miao et al. 2023).

PC dysfunction and detachment emerge as central features of NVU disruption in glaucoma and TON (Alarcon-Martinez et al. 2020). Mechanical stress and inflammatory signals trigger aberrant activation of MMPs, leading to PC detachment and weakening their interactions with ECs (Alarcon-Martinez et al. 2022). This PC loss destabilizes the vasculature, increases BRB permeability, and amplifies inflammatory mediator infiltration (Huang 2020). Furthermore, the absence of functional PCs renders ECs more vulnerable to inflammatory cytokines, such as TNF-α and IL-1β, exacerbating BRB breakdown and vascular instability (Ju et al. 2023).

Reactive astrocytes and Müller cells further contribute to NVU dysfunction following BRB disruption. Reactive astrocytes secrete elevated levels of vascular endothelial growth factor (VEGF) and pro-inflammatory cytokines, degrading endothelial tight junction proteins such as ZO-1 and occludin, and promoting vascular leakage (Guttenplan et al. 2020; Shinozaki et al. 2023). Concurrently, reactive Müller cells amplify ECM remodeling by releasing MMPs and contribute to the formation of gliotic scars (Li et al. 2021). These scars not only act as physical barriers to tissue repair and axonal regeneration but also create a biochemically hostile microenvironment that further compromises RGC survival.

Microglia, the resident immune cells of the retina, play a dual role in NVU dysfunction (Miao et al. 2023). Pathological activation of microglia leads to the release of excessive ROS, pro-inflammatory cytokines, and chemokines, which directly damage ECs and PCs while further activating glial cells and recruiting systemic immune cells (Liu et al. 2024). This excessive activation initiates a self-perpetuating inflammatory loop within the NVU, further destabilizing the retinal microenvironment and exacerbating neurodegeneration.

These pathological changes underscore the complexity of NVU dysfunction in glaucoma and TON. PC loss, glial cell reactivity, and microglial activation collectively drive BRB destabilization and create a retinal microenvironment that is hostile to neuroprotection and regeneration. Elucidating these NVU-specific pathological mechanisms offers critical insights into the development of targeted therapeutic strategies aimed at preserving BRB integrity and restoring NVU function to mitigate retinal neurodegeneration.

## Immune infiltration and inflammatory regulation: a turning point for RGC fate

**Fig. 2:**
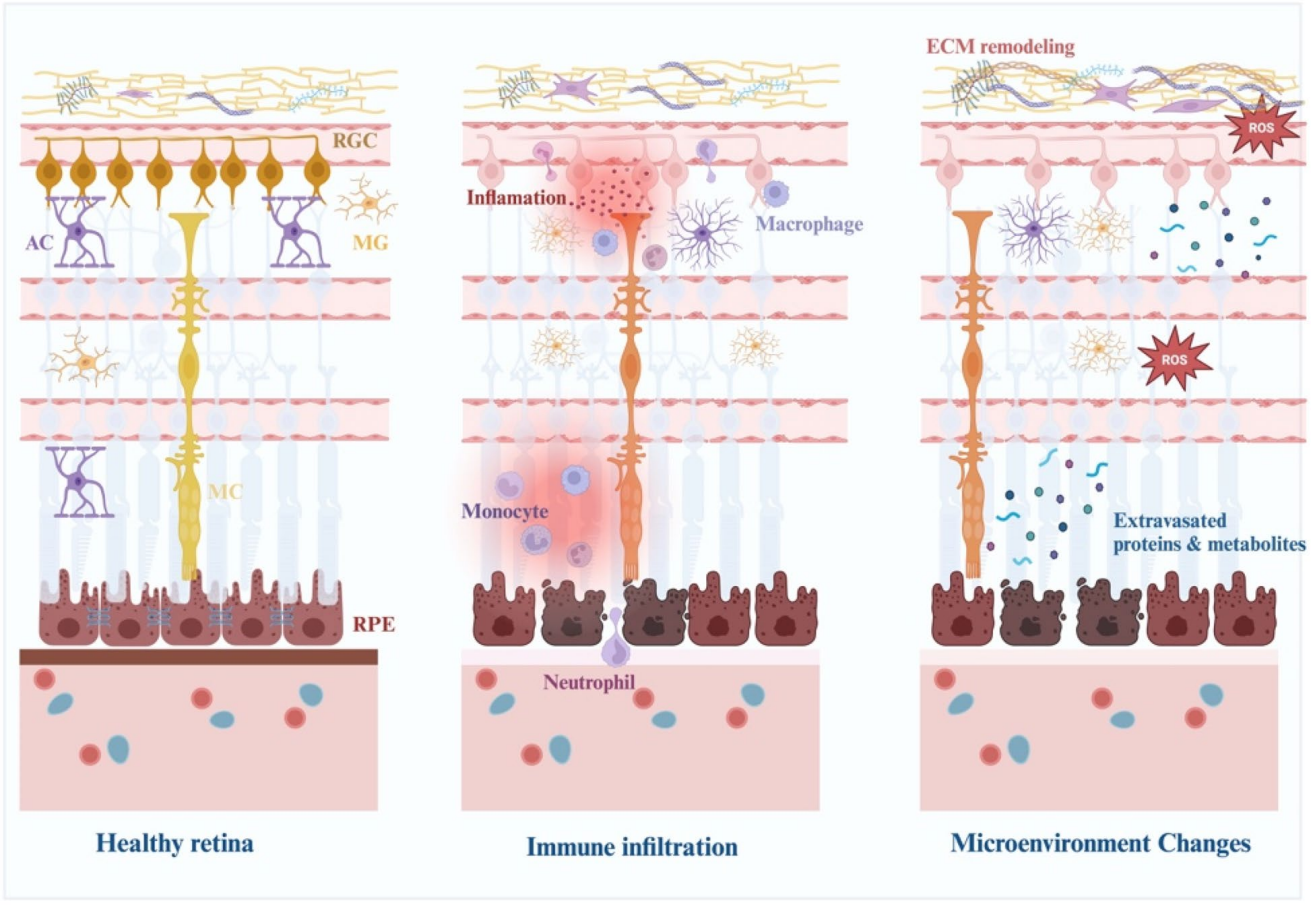
Progression of retinal damage: from healthy to pathological microenvironment. Healthy retina (Left panel): The retina under normal conditions exhibits an intact structure, including retinal ganglion cells (RGCs), astrocyte (ACs), Müller glia (MG), and retinal pigment epithelial (RPE) cells. These elements maintain a balanced microenvironment, supported by proper nutrient transport and immune privilege, essential for neuronal and vascular integrity. Immune infiltration (Middle panel): Retinal damage induces inflammation, characterized by immune cell infiltration, including neutrophils, monocytes, and macrophages. This process disrupts the BRB and activates glial cells, contributing to the release of inflammatory cytokines, chemokines, and reactive oxygen species (ROS), exacerbating tissue damage. Microenvironment changes (Right panel): Chronic inflammation leads to extracellular matrix (ECM) remodeling, ROS accumulation, and the extravasation of proteins and metabolites into the retinal tissue. These changes destabilize the retinal microenvironment, impairing neuronal survival and repair processes, and further promoting neurodegeneration

The breakdown BRB is a defining event in the pathogenesis of retinal neurodegenerative diseases such as glaucoma and TON (Alarcon-Martinez et al. 2023). BRB disruption profoundly alters the immune microenvironment of the retina, permitting systemic immune cells—such as neutrophils, macrophages, and lymphocytes—to infiltrate retinal tissues. This infiltration undermines the tightly regulated immune homeostasis critical for retinal function, initiating a cascade of inflammatory responses that accelerate RGC degeneration and hinder their regenerative potential (Fig. 2) (Au and Ma 2022; Passino et al. 2024).

Infiltrating immune cells release pro-inflammatory cytokines, including TNF-α, IL-1β, and IL-6, which amplify the inflammatory milieu and compromise the structural and functional integrity of ECs (Ouyang et al. 2022). These cytokines downregulate tight junction proteins such as occludins and claudins, thereby perpetuating vascular permeability and facilitating further immune cell recruitment (Abdelrahman et al. 2022). The chronic inflammation resulting from this cascade creates a hostile microenvironment for RGCs, characterized by heightened oxidative stress, increased apoptosis, and impaired axonal repair (Benhar et al. 2016). For example, while IL-6 may have protective roles in certain immune contexts, its prolonged activation can suppress RGC axon regeneration via interactions with STAT3 signaling pathways (Leibinger et al. 2016).

Neutrophils are among the earliest immune responders following BRB breakdown (Benhar et al. 2023). These cells exacerbate retinal damage by releasing ROS and proteases such as MMPs, which degrade ECM components and tight junctions (Baudouin et al. 2021). This damage destabilizes vascular integrity, further amplifying oxidative stress and inflammation, and contributing to RGC degeneration (Passino et al. 2024). Similarly, macrophages, which typically play reparative roles under physiological conditions, often shift to a pro-inflammatory phenotype in response to pathological stress (Sterling et al. 2024). These activated macrophages secrete cytokines and growth factors that sustain chronic inflammation and create a toxic microenvironment that compromises RGC survival (Conedera et al. 2024).

Emerging evidence reveals that the RPE serves as a gateway for monocyte trafficking into the retina, mirroring the role of the choroid plexus epithelium in the brain (Peng et al. 2024). Following retinal injury, such as ONC, the RPE upregulates adhesion molecules like VCAM-1, enabling monocyte recruitment (Benhar et al. 2016). These monocytes initially accumulate in the RPE before migrating into retinal tissues, where they modulate the immune microenvironment. Interestingly, the ocular milieu can induce neuroprotective phenotypes in infiltrating monocytes, suggesting their dual role in neuroinflammation and neuroprotection (Paschalis et al. 2019; Xie et al. 2022). However, disrupting VCAM-1-mediated monocyte infiltration biases the local cytokine profile toward a pro-inflammatory state, reducing the retina’s capacity for repair and underscoring the complexity of immune-epithelial interactions in retinal degeneration (Benhar et al. 2016).

The interplay between infiltrating immune cells and resident retinal cells, including ECs and glia, intensifies the inflammatory response (Benhar et al. 2023; Wang et al. 2024). ECs, when exposed to pro-inflammatory signals, adopt a pro-thrombotic and pro-inflammatory phenotype, upregulating adhesion molecules such as ICAM-1 and VCAM-1 to recruit and anchor leukocytes to the retinal vasculature (Zhang et al. 2025). This feedback loop perpetuates BRB dysfunction, exacerbating immune infiltration and further destabilizing the retinal microenvironment.

Ultimately, the combined effects of immune cell infiltration and chronic inflammation transform the retinal microenvironment into a pro-degenerative state (Pfeiffer et al. 2020). This hostile landscape drives RGC apoptosis, destabilizes vascular integrity, and severely limits regenerative capacity (Soto et al. 2019). These insights highlight the importance of therapeutic strategies targeting inflammatory pathways, immune-epithelial interactions, and BRB stabilization to mitigate RGC loss and promote neuroprotection. The following section will explore how glial activation contributes to the pathological landscape shaped by BRB disruption.

## Glial cells: catalysts in the fate of RGCs

The activation of glial cells, including microglia, Müller cells, and astrocytes, plays a pivotal role in amplifying inflammatory responses during BRB disruption (Zhang et al. 2024b). Under pathological conditions, these glial cells undergo reactive changes that destabilize the retinal microenvironment, profoundly influencing the survival and regenerative potential of retinal RGCs (Table 1)(London et al. 2013).

**Table 1:** Comparative Analysis of Gliosis and Inflammatory Mechanisms in Glaucoma and Traumatic Optic Neuropathy

	**Glaucoma**	**Traumatic Optic Neuropathy**
Triggering Factor	Chronic elevated intraocular pressure (IOP) causing mechanical stress	Acute trauma leading to mechanical and ischemic damage to the optic nerve
Müller Cell Activation	Mechanism: Activation of mGluR1, inhibition of Kir4.1 channels Effects: Release of neurotoxic glutamate, reactive gliosis, and increased expression of glial fibrillary acidic protein (GFAP)	Mechanism: Induction of oxidative stress and metabolic dysregulation Effects: Rapid release of pro-inflammatory cytokines (IL-6, TNF-α) and disruption of potassium buffering
Microglia Activation	Mechanism: ATP release, TLR4 signaling, and increased complement activation Effects: Chronic inflammation, release of TNF-α, IL-1β, and ROS	Mechanism: Activation by DAMPs (damage-associated molecular patterns) and axonal debris Effects: Phagocytosis of RGC debris, exacerbation of acute inflammation
Astrocyte Activation	Mechanism: Chronic stress triggers extensive ECM remodeling, upregulation of pro-inflammatory cytokines and disruption of tight junction proteins Effects: Leads to breakdown of BRB, heightened inflammatory responses, and exacerbation of neuroinflammatory cascades, ultimately contributing to a hostile retinal microenvironment	Mechanism: Response to ischemia and axonal disruption Effects: Release of inflammatory cytokines and chemokines (CXCL10, MCP-1) driving monocyte recruitment
Role of Gliosis in Pathology	Progressive RGC degeneration due to chronic excitotoxicity	Acute RGC death and axonal loss due to neurotoxic and inflammatory cascades

Microglia, as the first responders to retinal injury, are central to orchestrating inflammatory responses (Borst et al. 2021). Upon activation, microglia secrete pro-inflammatory cytokines, such as TNF-α and IL-1β, as well as chemokines that propagate inflammation and exacerbate BRB disruption (Madore et al. 2020). While microglial activation is critical for responding to injury, overactivation can result in chronic inflammation and neurodegeneration by inducing RGC apoptosis through cytokine-mediated pathways (Hilla et al. 2017). Conversely, microglia also exhibit protective functions, such as clearing cellular debris and mitigating neutrophil-induced vascular damage, underscoring their dual, context-dependent roles in retinal pathophysiology (Passino et al. 2024).

**Fig. 3:**
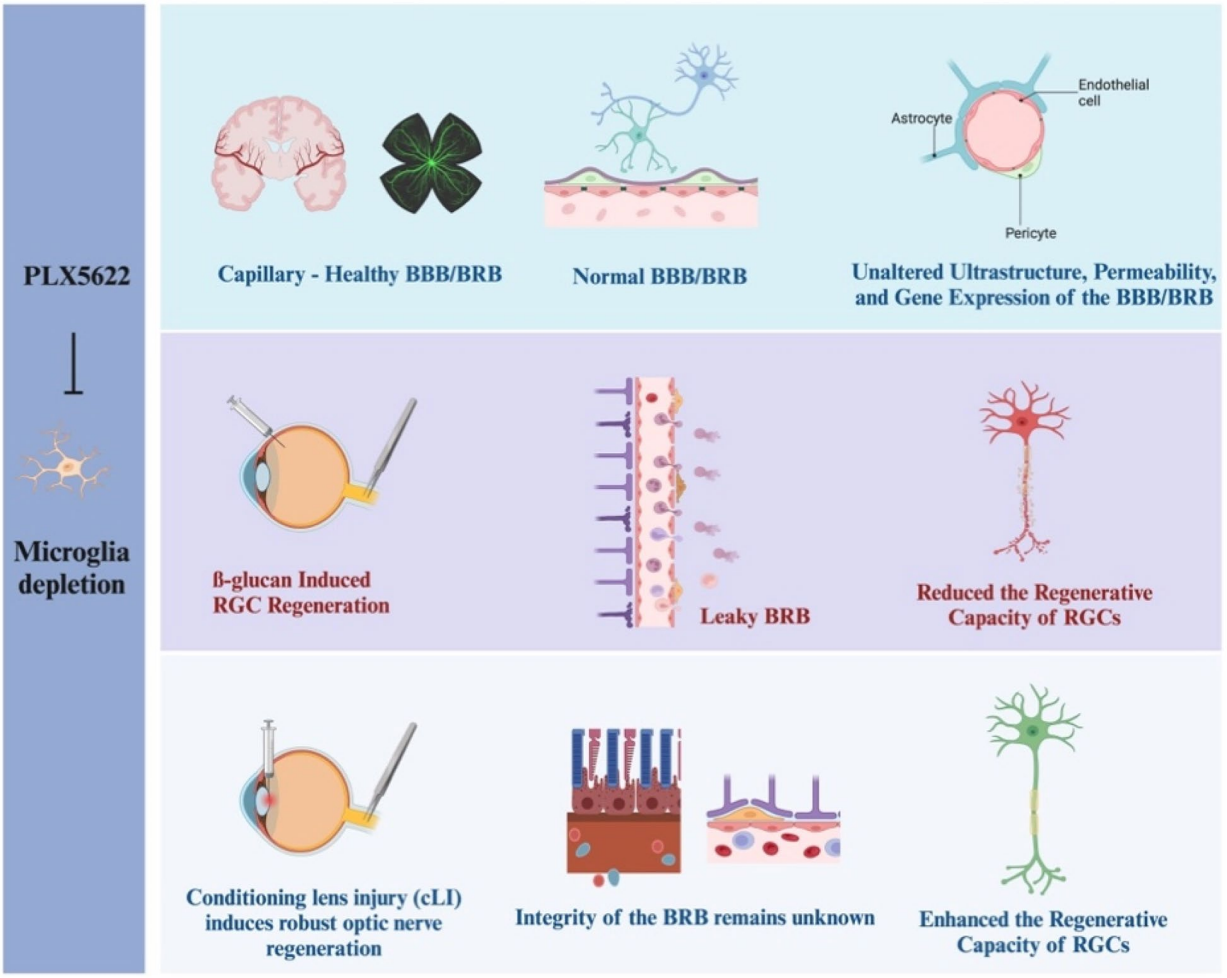
The dual roles of microglial depletion in RGC regeneration and BRB integrity. Healthy BBB/BRB (Top row): Microglial depletion using PLX5622 does not alter the ultrastructure, permeability, or gene expression of the blood–brain barrier (BBB) or BRB under healthy conditions. The endothelial cells, pericytes, and astrocytes maintain their integrity and functional interactions, preserving the neurovascular unit in both the brain and retina. Pathological BRB with compromised RGC regeneration (Middle row): Under conditions of β-glucan-induced RGC regeneration, microglial depletion leads to leaky BRB, as demonstrated by increased vascular permeability and infiltration of immune cells. This compromised BRB correlates with reduced regenerative capacity of RGCs, indicating the importance of microglia in maintaining a supportive environment for axonal repair under certain pathological states. Conditioning lens injury and RGC regeneration (Bottom row): In the conditioning lens injury (CLI) model, which induces robust optic nerve regeneration, microglial depletion paradoxically enhances the regenerative capacity of RGCs. The integrity of the BRB in this context remains unclear, indicating potential changes in the immune system and surrounding environment that make regeneration more favorable

Recent evidence has highlighted the intricate duality of microglial function within the retinal microenvironment (Fig. 3) (Yun-Jia et al. 2021; Zou et al. 2024). While excessive activation contributes to chronic inflammation and neuronal damage, microglia are also essential for maintaining homeostasis by regulating immune responses and preserving vascular integrity (Au and Ma 2022). Pharmacological depletion of microglia using colony-stimulating factor 1 receptor (CSF1R) inhibitors, such as PLX5622, has shed light on the complexity of their roles. Interestingly, studies suggest that microglia are not essential for maintaining the structural or functional integrity of the BRB or blood–brain barrier (BBB) under healthy conditions (Profaci et al. 2024). This finding underscores the inherent resilience of these barriers in the absence of microglia under physiological states.

However, in pathological contexts, microglial activity takes on a more nuanced role (Hilla et al. 2017). Microglial depletion has been associated with increased BRB permeability, heightened levels of pro-inflammatory neutrophils, and reduced RGC regeneration in inflammatory environments (Liu et al. 2024). These findings underscore the critical role of preserving vascular integrity for neuronal repair and regeneration. Paradoxically, in the context of conditional lens injury (cLI)—a model that enhances RGC regeneration through immune-mediated mechanisms—microglial depletion has been shown to enhance axon regeneration, with some axons extending into the brain (Feng et al. 2023). This raises compelling questions about whether BRB permeability changes induced by microglial depletion facilitate immune cell infiltration and indirectly enhance regeneration. Elucidating the interplay between vascular stability, immune modulation, and microglial activity remains a key area for further investigation.

Müller cells, which span the entire thickness of the retina, are crucial for maintaining retinal homeostasis but become reactive under stress (Bringmann et al. 2006; Yin et al. 2019). When activated, Müller cells release VEGF, MMPs, and inflammatory mediators that exacerbate vascular permeability and drive ECM remodeling (Wang 2015). Reactive Müller cells also contribute to gliotic scar formation, which physically and biochemically impedes axonal regeneration (Balzamino et al. 2024). This dual role highlights the complexity of Müller cell activation: while attempting to stabilize the retinal environment, their reactive state often creates barriers to neuroregeneration.

Similarly, astrocytes, another key glial component, undergo reactive changes following BRB disruption (Lin et al. 2020). Reactive astrocytes release ECM components and cytokines that promote chronic inflammation, further destabilizing the BRB and contributing to the formation of glial scars (Guttenplan et al. 2020). While these scars may initially protect against further damage, their persistence impedes axonal regrowth and sustains an inflammatory microenvironment (Liu et al. 2021). Together, the activation of microglia, Müller cells, and astrocytes forms a self-perpetuating cycle of inflammation and gliosis that accelerates RGC degeneration and hinders regenerative efforts.

The cascade of immune infiltration, glial activation, and chronic inflammation triggered by BRB disruption underscores the complex mechanisms shaping RGC fate in glaucoma and TON. These interconnected processes not only exacerbate neuronal degeneration but also establish formidable barriers to regeneration, underscoring the urgency of developing therapeutic strategies that target glial cell activity, mitigate inflammation, and preserve BRB integrity to promote RGC survival and recovery.

## Impacts of BRB disruption on the retinal microenvironment

The BRB plays a pivotal role in maintaining retinal microenvironmental homeostasis (Kim et al. 2024). Its disruption not only triggers immune cell infiltration and inflammatory responses but also induces ECM remodeling, exacerbates oxidative stress, and disrupts metabolic balance (O’Leary and Campbell 2023). These changes drive pathological alterations in retinal tissues, worsening RGC degeneration and impeding their regeneration.

### Pathological effects of ECM remodeling

BRB disruption promotes immune cell infiltration and activates MMPs, significantly altering the normal structure of the ECM (Zhang et al. 2024a). MMPs degrade critical ECM components, such as collagen and laminin, weakening the supportive structure required for RGC survival and interrupting regenerative signaling (Fu et al. 2022). Abnormal ECM remodeling is often accompanied by fibrosis and glial scar formation, which not only physically block RGC axonal regeneration but also alter retinal mechanical properties, further aggravating the hostile retinal microenvironment (Reinhard et al. 2021).

### Exacerbation of oxidative stress

Oxidative stress is markedly increased following BRB disruption (Tisi et al. 2021). The accumulation of ROS directly damages RGCs and other retinal cells, while inducing mitochondrial dysfunction that accelerates apoptosis or necrosis (Wen et al. 2021; Zhu et al. 2024). ROS generation synergizes with MMP activation, intensifying ECM degradation and further destabilizing the retinal microenvironment (Kowluru and Shan 2017). Oxidative stress also activates inflammatory pathways such as NF-κB, perpetuating chronic inflammation and weakening retinal repair mechanisms. Antioxidant therapies, such as N-acetylcysteine (NAC) and edaravone, show potential for mitigating ROS production and protecting RGCs, offering a promising strategy for retinal pathologies (Guo et al. 2022).

### Interactions between metabolic dysregulation and retinal pathology

Metabolic dysregulation constitutes a major component of retinal pathology following BRB disruption (Gabrielle 2022). Lipid metabolic imbalances, including cholesterol accumulation, abnormal polyunsaturated fatty acid (PUFA) metabolism, and intensified lipid peroxidation, are key contributors to RGC degeneration (Yang et al. 2020). The release of pro-inflammatory cytokines and ROS resulting from BRB disruption may drive lipid metabolic imbalances, further compromising the stability of RGC membranes (Zhou et al. 2024b). Additionally, the outer BRB, formed by RPE, plays an essential role in lipid transport and metabolism (Hansman et al. 2025). BRB dysfunction disrupts these processes, leading to cholesterol deposition and metabolic imbalance (Tong et al. 2023).

Glucose metabolic dysregulation also exacerbates retinal pathology (Léveillard and Sahel 2017). The retina’s high energy demands make it vulnerable to metabolic imbalances, impairing normal RGC function and further hindering their regenerative potential (Zhou et al. 2024c). Interactions between lipid and glucose dysregulation and oxidative stress create a vicious cycle, significantly worsening retinal damage (Joyal et al. 2016; Harder et al. 2020).

The multifaceted impacts of BRB disruption on the retinal microenvironment highlight its critical role in regulating RGC fate. Further investigation into its interactions with ECM remodeling, oxidative stress, and metabolic dysregulation will not only shed light on the pathogenesis of glaucoma and TON but also guide the development of targeted therapeutic strategies.

## Comparative analysis of BRB disruption mechanisms in retinal diseases

The breakdown of the BRB is a shared pathological hallmark in a variety of retinal diseases, including diabetic retinopathy (DR), age-related macular degeneration (AMD), glaucoma, and TON (Alarcon-Martinez et al. 2023; Liao et al. 2024; Kaufmann and Han 2024). Despite this common feature, the molecular and cellular mechanisms underlying BRB dysfunction differ significantly depending on the disease context, reflecting diverse pathological drivers (O’Leary and Campbell 2023).

In DR, BRB disruption is driven by chronic metabolic stress caused by prolonged hyperglycemia (Wimmer et al. 2019). This results in the accumulation of advanced glycation end-products (AGEs) and activation of protein kinase C (PKC) signaling pathways, which destabilize tight junction proteins such as occludins and claudins, thereby increasing vascular permeability (Li et al. 2024b). Chronic low-grade inflammation and oxidative stress further compromise the inner BRB, resulting in endothelial cell damage, vascular leakage, and retinal edema (Antonetti et al. 2021). A defining feature of DR is the pathological overexpression of VEGF, which promotes abnormal angiogenesis and exacerbates inflammation (Simó and Hernández 2023). The newly formed vessels are highly permeable and fragile, compounding BRB failure and retinal neurodegeneration (Simó et al. 2020). These processes reflect the chronic metabolic nature of DR pathology, where sustained dysregulation of vascular homeostasis drives progressive BRB dysfunction (Monickaraj et al. 2023).

By contrast, in glaucoma, BRB disruption is primarily a consequence of sustained IOP, which imposes mechanical stress on retinal ECs and PCs (Pang and Clark 2020; Baudouin et al. 2021). This mechanical strain damages tight junction proteins and activates stress-responsive signaling pathways, including MAPK and NF-κB (Liu et al. 2022; Ju et al. 2023). These pathways initiate inflammatory cascades, amplifying BRB breakdown and microvascular instability (Stepp and Menko 2021). Unlike the chronic metabolic stress observed in DR, glaucoma pathology is defined by acute mechanical insults that disrupt the structural and functional integrity of the inner BRB, leading to vascular dysfunction and exacerbating RGC degeneration.

In AMD, the pathology centers on the outer BRB, where RPE are compromised by chronic oxidative stress, lipid accumulation, and excessive activation of the complement system (Mitchell et al. 2018; Sharma et al. 2020). Complement components such as C3a and C5a promote RPE apoptosis and tight junction degradation, resulting in the breakdown of the outer BRB (Kim et al. 2021). In advanced stages of AMD, choroidal neovascularization (CNV) penetrates the RPE layer, causing severe vascular leakage and neuroinflammation, which ultimately accelerates retinal degeneration (Mettu et al. 2021). This contrasts with TON, where acute immune responses dominate. In TON, the RPE also serves as a gateway for monocyte trafficking into the retina, mediated by the upregulation of adhesion molecules like ICAM-1 and VCAM-1 (Benhar et al. 2016). However, the oBRB disruption in TON is characterized by acute inflammatory infiltration rather than the chronic oxidative and complement-mediated damage observed in AMD (Taylor et al. 2021).

These disease-specific patterns of BRB disruption illustrate the diversity of pathological drivers across retinal diseases. DR highlights the impact of chronic metabolic stress on vascular stability, glaucoma underscores the consequences of mechanical stress and inflammatory cascades, and AMD emphasizes oxidative and complement-mediated damage to the outer BRB. TON, with its acute inflammatory responses, further demonstrates the variability of BRB dysfunction in retinal diseases. Understanding these distinct yet overlapping mechanisms not only elucidates disease progression but also provides a foundation for developing targeted therapeutic strategies tailored to each condition's unique pathological drivers.

## Conclusions and perspectives

The integrity of the BRB is fundamental for maintaining retinal homeostasis and supporting RGC survival. However, its disruption is a central pathological event in glaucoma and TON, initiating cascades of immune infiltration, glial activation, oxidative stress, and metabolic dysregulation. These interconnected mechanisms transform the retinal microenvironment into a hostile landscape that exacerbates neurodegeneration and significantly hinders neural regeneration. This review has highlighted the structural and functional characteristics of the BRB, the role of the NVU in maintaining its integrity, and the mechanisms underlying its breakdown in glaucoma and TON. By integrating recent findings, we provide a comprehensive understanding of how BRB disruption contributes to RGC degeneration and how these insights can inform therapeutic strategies.

Future research should focus on resolving several key challenges. First, understanding the distinct molecular and cellular mechanisms driving BRB dysfunction in glaucoma and TON is essential for developing targeted interventions. Advanced technologies, such as single-cell and spatial transcriptomics, will allow deeper insights into cell-type-specific changes and spatial interactions within the NVU and retinal microenvironment. These tools can illuminate the complex interplay between immune cells, glial cells, and vascular components in disease progression. Second, bridging the gap between preclinical models and human pathology remains critical for translational success. Improved animal models that better replicate the chronic and acute pathologies of glaucoma and TON, coupled with patient-derived organoids, may accelerate the validation of therapeutic strategies.

Therapeutically, restoring BRB integrity and modulating the immune microenvironment hold promise for mitigating RGC loss. Strategies targeting inflammatory signaling pathways, oxidative stress, and metabolic dysregulation are emerging as viable avenues for intervention. In particular, therapeutic approaches that leverage the dual roles of immune cells, such as monocytes and microglia, to promote neuroprotection while minimizing chronic inflammation warrant further exploration.

Finally, the complexity of BRB disruption underscores the need for integrative and multidisciplinary approaches. Combining gene therapy, small-molecule drugs, and advanced biomaterials for localized drug delivery could enhance therapeutic efficacy while minimizing systemic side effects. Collaborative efforts between basic researchers and clinicians will be critical to translating these innovations into effective treatments. By addressing these challenges, future studies have the potential to not only deepen our understanding of BRB-related mechanisms in glaucoma and TON but also pave the way for transformative therapies that restore vision and improve patient outcomes.

## Data Availability

Not applicable.

## Abbreviations

RGCs: Retinal ganglion cells
TON: Traumatic optic neuropathy
BRB: Blood-retinal barrier
ECs: Endothelial cells
PCs: Pericytes
RPE: Retinal pigment epithelial
NVU: Neurovascular unit
ONC: Optic nerve crush
ZO-1: Zonula occludens-1
PDGF-β: Platelet-derived growth factor- beta
TGF-β: Transforming growth factor-beta
AQP4: Aquaporin-4
IOP: Intraocular pressure
ECM: Extracellular matrix
NETs: Neutrophil extracellular traps
ROS: Reactive oxygen species
MMP-3: Matrix metalloproteinase-3
I/R: Ischemia-reperfusion
MMPs: Matrix metalloproteinases
SDF-1α: Stromal cell-derived factor-1α
CXCR4: C-X-C chemokine receptor type 4
VEGF: Vascular endothelial growth factor
CSF1R: Colony-stimulating factor 1 receptor
cLI: Conditional lens injury
GFAP: Glial fibrillary acidic protein
DAMPs: Damage-associated molecular patterns
NAC: N-acetylcysteine
PUFA: Polyunsaturated fatty acid
DR: Diabetic retinopathy
AMD: Age-related macular degeneration
AGEs: Advanced glycation end-products
PKC: Activation of protein kinase C
CNV: Choroidal neovascularization
